# PCTAIRE1 regulates p27 stability, apoptosis and tumor growth in malignant melanoma

**DOI:** 10.18632/oncoscience.86

**Published:** 2014-10-05

**Authors:** Teruki Yanagi, John C. Reed, Shu-ichi Matsuzawa

**Affiliations:** ^1^ Sanford-Burnham Medical Research Institute, La Jolla, CA, USA

**Keywords:** PCTAIRE1, p27, apoptosis, melanoma

## Abstract

PCTAIRE1 is a cyclin-dependent kinase family protein that has been implicated in spermatogenesis. Although we recently revealed the function of PCTAIRE1 in tumorigenesis of epithelial carcinoma cells, its tumorigenic function in melanoma remains unclear. Interrogation of the Oncomine database revealed that malignant melanoma showed up-regulation of PCTAIRE1 mRNA compared to normal skin and benign melanocytic nevus tissues. In the melanoma cell lines A2058 and SK-MEL-28, *PCTAIRE1* gene knockdown using siRNA or shRNA diminished melanoma cell proliferation as assessed by cellular ATP levels, cell counting and clonogenic assays. Moreover, FACS analyses of annexin V-PI staining and DNA content showed that PCTAIRE1 knockdown caused apoptosis in A2058 cells. In contrast, PCTAIRE1 does not appear to be involved in the proliferation of immortalized human keratinocyte HaCaT cells. Depletion of PCTAIRE1 by siRNA/shRNA led to p27 accumulation in melanoma cells but not HaCaT cells. In tumor xenografts of melanoma A2058 cells, conditional knockdown of PCTAIRE1 restored p27 protein expression and suppressed tumor growth. Our findings reveal a crucial role for PCTAIRE1 in regulating p27 protein levels and tumor growth in melanoma cells, suggesting that PCTAIRE1 could provide a target for melanoma treatment.

## INTRODUCTION

Melanoma is a highly aggressive skin cancer that originates from melanocytes, and its incidence has been rising substantially over the past decades [1]. Predictions indicate that in 2014 there will be approximately 76,100 new cases of invasive melanoma diagnosed in the United States and 9,710 deaths from the disease [2]. Predisposition to melanoma can be influenced by an individual's genetic background, pigmentation status and exposure to ultraviolet light [3]. Multiple cellular pathways have been implicated in melanomagenesis, ranging from signal transduction to developmental and transcriptional pathways as well as cell cycle deregulation [4]. Although melanoma can be treated by surgical intervention, radiation, chemotherapy and a combination of these therapies, the prognosis of patients with metastatic disease is poor. To obtain more effective and less toxic therapies that improve survival, better characterization of the molecular mechanisms involved in melanoma pathogenesis and identification of new drug targets is of critical importance.

PCTAIRE1 (also known as PCTK1 and cyclin-dependent kinase 16 (Cdk16)) is a member of the PCTAIRE family, a group of kinases related to the Cdk family [5]. PCTAIRE1 is ubiquitously expressed, with high levels in the brain and testis [6]. A physiological function for PCTAIRE1 was suggested by findings with neurons from mutant PCT-1 nematodes, which show altered axonal vesicle transport [7], while mammalian PCTAIRE1 was implicated by gene knockout studies in mice to be essential for spermatogenesis [8], regulation of intracellular vesicles [9, 10] and neurite outgrowth [11]. Although PCTAIRE1 contains a motif similar to the cyclin binding sites found in Cdk family members [5], the mechanisms for its activation are still unclear [5, 11]. PCTAIRE1 is activated by interactions with cyclin Y [8], while PCTAIRE1 binding with a Cdk5 activator does not activate its kinase activity [12]. The PCTAIRE1 substrate phosphorylation consensus sequence is currently undefined, although PCTAIRE1 is known to bind and phosphorylate Ser569 of the vesicular transport protein NSF [9] and phosphorylate myelin basic protein *in vitro* [13]. PCTAIRE1 also interacts with the COPII complex involved in the export of secreted proteins from the endoplasmic reticulum [10]. Recently, we presented evidence that PCTAIRE1 plays an indispensable role in the proliferation of some types of epithelial carcinoma cells [14] in that PCTAIRE1-depleted carcinoma cells showed apoptosis with mitotic arrest associated with centrosome dysregulation. Moreover, we showed that PCTAIRE1 directly binds p27 and promoted phosphorylation at Ser10, which led to degradation of this tumor suppressor.

In this study we investigated the role of PCTAIRE1 in tumorigenesis of melanoma cells and found that PCTAIRE1-depleted melanoma cells show suppressed cell growth with apoptosis, and PCTAIRE1 knockdown led to accumulation of the tumor suppressor p27. In tumor xenografts, conditional knockdown of PCTAIRE1 restored p27 protein expression and suppressed tumor growth. Our study reveals an indispensible role for PCTAIRE1 in melanoma cells, suggesting that this kinase may provide a novel target for the future development of melanoma therapeutics.

## RESULTS

### PCTAIRE1 is overexpressed in malignant melanoma

**Figure 1:**
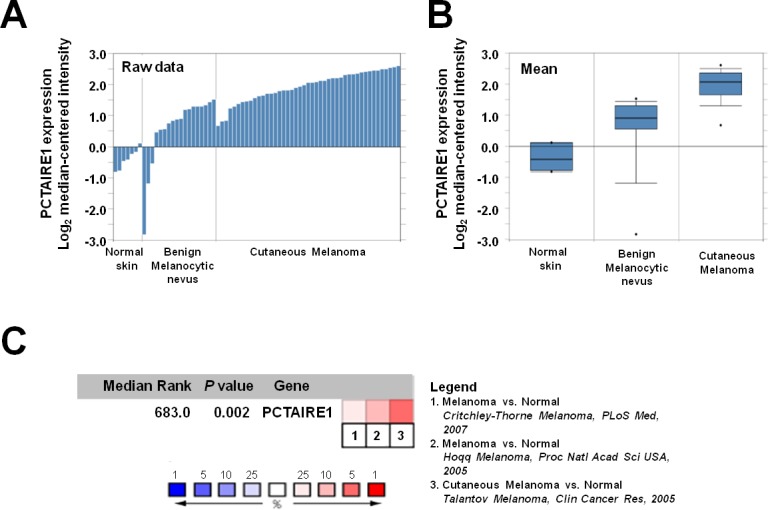
PCTAIRE1 expression is up-regulated in melanomas (A, B) Microarray datasets were accessed using the Oncomine database (http://www.oncomine.com). PCTAIRE1 expression (as log2 median-centered intensity) for normal skin, benign melanocytic nevus and malignant melanoma is shown either as raw data (A) or as the mean (B). In the chart showing the mean, dots indicate extreme data values. Melanoma vs. Normal: Fold change = 5.027, P = 3.98 × 10^−9^, Melanoma vs. Melanocytic nevus: Fold change = 2.504, P = 4.55 × 10^−5^. (C) Meta-analysis of PCTAIRE1 gene expression profiling where colored squares indicate the median rank for PCTAIRE1 (vs. Normal skin) across each analysis. PCTAIRE1 ranks in the top 10% in two of three analyses. The P value is given for the median-rank analysis.

To determine whether PCTAIRE1 expression is associated with malignant melanoma, we interrogated the Oncomine database. The study by Talantov and colleagues was initially selected, as this dataset includes a substantial number of tissue samples from normal skin (n = 7) and benign melanocytic nevi (n = 18), as well as a large number of malignant melanoma samples (n = 45; Figure 1A and B) [15]. This dataset showed that PCTAIRE1 mRNA levels were significantly higher in melanoma tumors compared to normal or benign melanocytic nevus tissues (see legend for individual P values). Data from other studies that included normal tissue samples showed similar results (Figure 1C). Overall, when all the studies containing information regarding PCTAIRE1 expression levels were combined, PCTAIRE1 was identified as one of the genes that is up-regulated in melanoma relative to normal skin tissue.

### Knockdown of PCTAIRE1 regulates melanoma cell growth and proliferation

**Figure 2:**
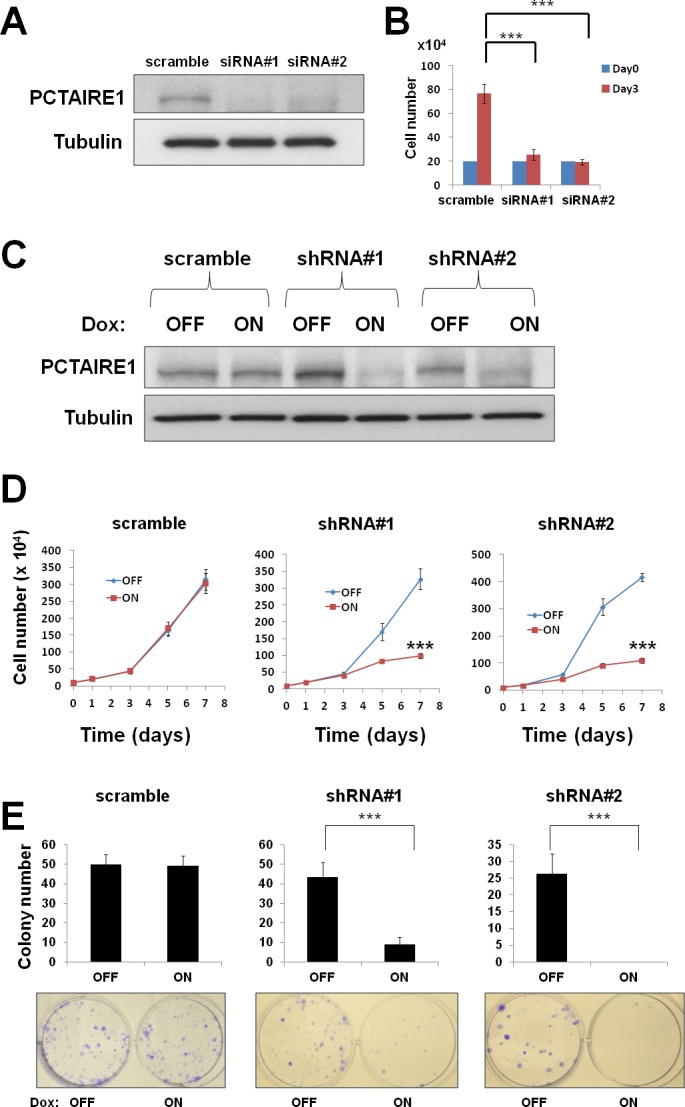
PCTAIRE1 knockdown diminished melanoma A2058 cell growth (A) A2058 cells were transfected with scrambled RNA or one of two different siRNAs targeting PCTAIRE1 mRNA (siRNA#1, #2). After 72 hours, cell lysates were analyzed by immunoblotting. (B) To measure cell growth, 2.0 × 10^5^ cells transfected with the indicated siRNAs were seeded onto 60 mm diameter plates. After 72 hours, the numbers of cells were counted. *** p < 0.001 (C) Protein lysates were generated from A2058 cells stably containing inducible shRNAs targeting PCTAIRE1 (shRNA#1, #2) and scramble control cultured for 72 hours with or without 100 ng/ml doxycycline, and analyzed by immunoblotting. (D) A2058 cells (10.0 × 10^4^) stably containing inducible shRNAs were cultured in 60 mm diameter plates for 24 hours, then stimulated with (ON) or without (OFF) doxycycline. The numbers of cells were counted (mean ± SD; n = 3). *** p < 0.001 (E) A2058 cells stably containing inducible shRNAs were seeded at 300 cells per well in 60 mm dishes. After 24 hours, doxycycline was added. Colonies consisting of > 50 cells were enumerated on day 10. All data represent mean ± SD (n = 3). *** p < 0.001

**Figure 3:**
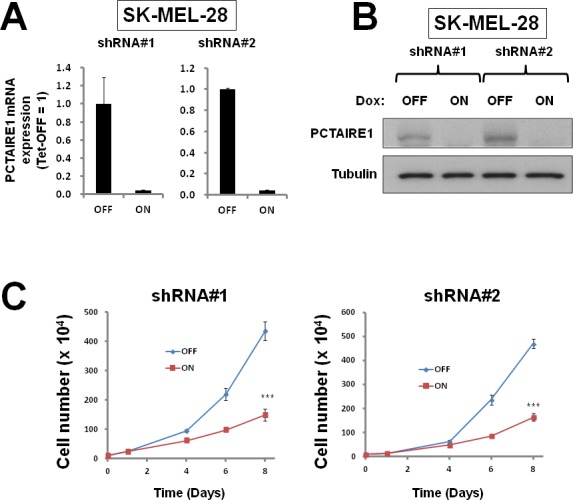
PCTAIRE1 knockdown diminished SK-MEL-28 cell growth (A) SK-MEL-28 cells stably containing inducible shRNAs targeting different sites on PCTAIRE1 mRNA (shRNA#1, #2) were cultured for 3 days with 100 ng/ml doxycycline (Dox). PCTAIRE1 mRNA levels were measured by qRT-PCR, with normalization relative to GADPH (mean ± SD; n = 2). (B) SK-MEL-28 cells with inducible shRNAs (#1, #2) were cultured for 3 days with (ON) or without (OFF) doxycycline. Protein lysates were generated and analyzed by immunoblotting. (C) SK-MEL-28 cells (5.0 × 10^4^) stably containing shRNAs were cultured for 24 hours, then stimulated with (ON) or without (OFF) doxycycline. The numbers of cells were counted (mean ± SD; n = 3). *** p < 0.001

**Figure 4:**
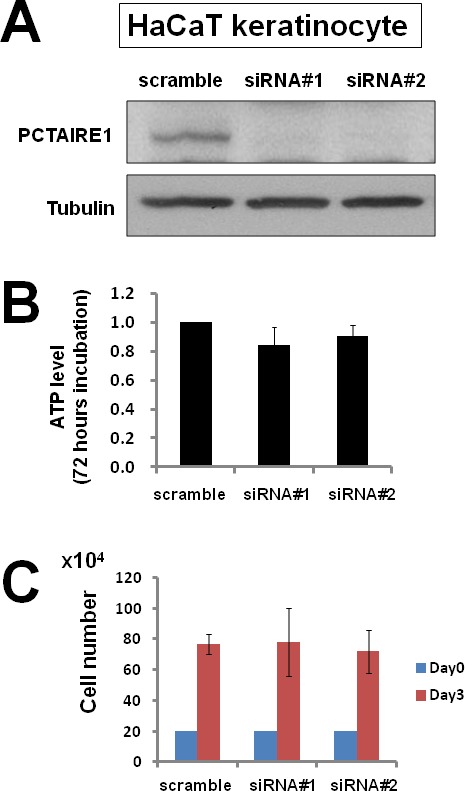
PCTAIRE1 knockdown did not diminish HaCaT keratinocyte growth (A) HaCaT keratinocytes were transfected with scrambled RNA or two different siRNAs targeting PCTAIRE1 (siRNA#1, #2). Three days after transfection, cell lysates were prepared and analyzed by immunoblotting. (B) HaCaT cells were transfected with siRNAs as indicated. After 3 days, cellular ATP levels were measured using Cell Titer Glo reagents (mean ± SD; n = 3). (C) To measure cell growth, 2.0 × 10^5^ cells transfected with the indicated siRNAs were plated. After 3 days, the number of cells was counted (mean ± SD; n = 3).

To investigate the effect of PCTAIRE1 knockdown in melanoma A2058 cells, we performed siRNA experiments using two siRNAs that target PCTAIRE1. Immunoblotting confirmed knockdown of protein levels by both PCTAIRE1 siRNAs tested (Figure 2A). Next, human melanoma A2058 cells were treated with negative control siRNAs or siRNAs targeting PCTAIRE1 and cell viability was assessed 3 days later. Three days after transfection, cultures of PCTAIRE1 knockdown A2058 cells showed reduced numbers of viable cells compared to control cell cultures (Figure 2B). Next, we used tetracycline-inducible shRNA vectors targeting PCTAIRE1 to assess the impact of PCTAIRE1 deficiency on the growth and survival of tumor cells. In A2058 cells that were stably infected with lentivirus expressing two different shRNAs targeting PCTAIRE1, reductions in PCTAIRE1 protein induced by the tetracycline analog doxycycline were documented by immunoblotting (Figure 2C). Compared to control cells (Tet-OFF), the growth rates of doxycycline-stimulated PCTAIRE1 knockdown A2058 cells (Tet-ON) were significantly diminished 5 days after seeding (Figure 2D). In clonogenic assays, the number of tumor cell colonies was significantly lower with PCTAIRE1 knockdown (Tet-ON) than for control cells (Tet-OFF) (Figure 2E). Similar results were obtained using other melanoma cell lines, such as SK-MEL-28 cells (Figure 3). We next assessed the PCTAIRE1 knockdown effect on skin keratinocytes. In the human immortalized keratinocyte cell line HaCaT, PCTAIRE1 knockdown did not affect cell growth as assessed by measurement of ATP levels and cell numbers (Figure 4). These results suggest that proliferation of malignant melanoma cells may be preferentially dependent on PCTAIRE1.

### Knockdown of PCTAIRE1 results in melanoma cell apoptosis

**Figure 5:**
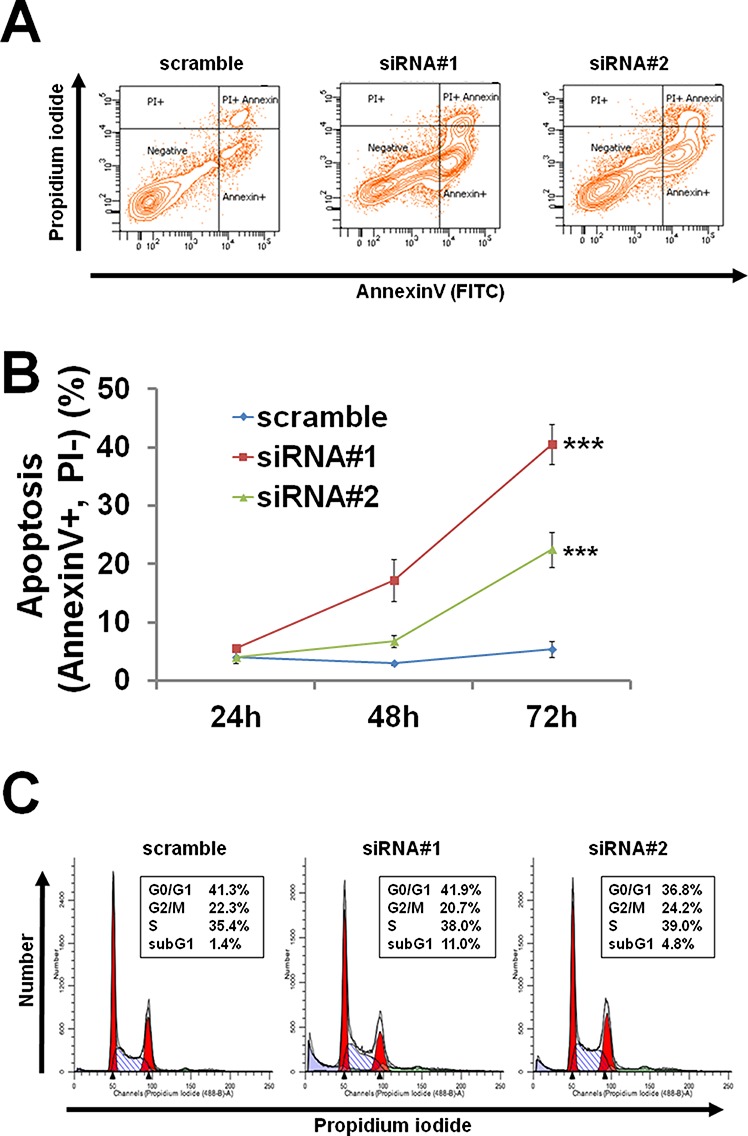
PCTAIRE1 knockdown resulted in apoptosis of A2058 cells (A, B) A2058 cells were transfected with the indicated siRNAs. Cells stained with annexin V and PI were assessed by FACS analyses at the indicated times. (A) Representative data at 72 hours after transfection are shown. (B) The percentage of apoptotic (annexin V positive and PI negative) cells is shown. All data represent mean ± SD (n = 3). *** p < 0.001 (C) A2058 cells transfected with siRNAs were cultured for 72 hours, followed by FACS analysis. Data represent relative DNA (propidium iodide fluorescence; x-axis) versus relative cell number (y-axis).

To examine the mechanisms of cell growth suppression by PCTAIRE1 knockdown, an annexin V-PI analysis was performed. Annexin V staining showed increases in apoptosis over time in cultures of PCTAIRE1 RNAi-treated cells (Figure 5A and B). Cell cycle analyses were also performed using FACS analysis of A2058 cells with PCTAIRE1 knockdown to assess DNA content, which allowed the percentages of 2N, 4N, polyploid (DNA content > 4N) and hypoploid cells to be quantified (Figure 5C). In cultures of PCTAIRE1 knockdown cells, the hypoploid apoptotic cell population (siRNA#1: 11.0%, siRNA#2: 4.8%) was increased, while PCTAIRE1 knockdown did not appreciably alter cell cycle distribution as the percentages of 2N, 4N, and S phase cells were similar between the PCTAIRE1-knockdown and control cells. These results suggest that in melanoma cells depletion of PCTAIRE1 results in apoptosis rather than cell cycle arrest.

### PCTAIRE1 regulates p27 stability

**Figure 6:**
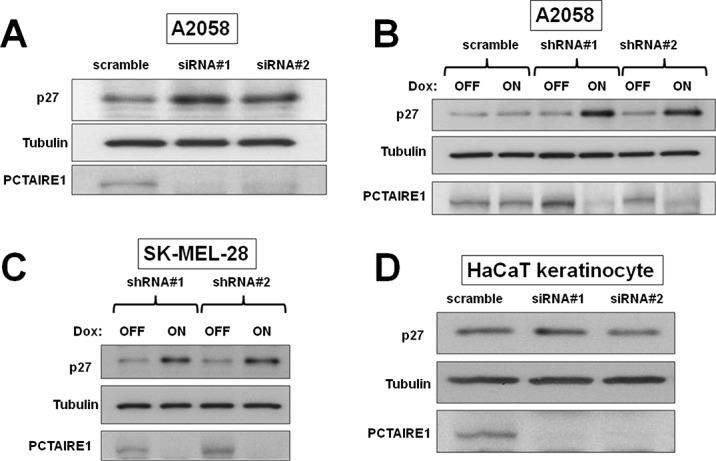
PCTAIRE1 knockdown leads to accumulation of p27 (A) A2058 cells were transfected with control RNA or two different siRNAs targeting PCTAIRE1. Three days after transfection, cell lysates were prepared and analyzed by immunoblotting. (B) A2058 cells stably containing inducible shRNAs targeting PCTAIRE1 (shRNA#1, #2) were cultured for 3 days with (ON) or without (OFF) 100 ng/ml doxycycline (Dox). Protein lysates were analyzed by SDS-PAGE/immunoblotting. (C) SK-MEL-28 cells with inducible shRNAs (#1, #2) were cultured for 3 days with (ON) or without (OFF) doxycycline (Dox). Protein lysates were analyzed by SDS-PAGE/ immunoblotting. (D) HaCaT keratinocytes were transfected with RNAs as indicated. After 3 days, cell lysates were prepared and analyzed by immunoblotting.

Since PCTAIRE1 plays an important role in p27 phosphorylation and degradation [14], we investigated the role of PCTAIRE1 in p27 stability of melanoma cells. PCTAIRE1 knockdown by siRNAs or Tet-inducible shRNAs up-regulated p27 protein levels in both the A2058 and SK-MEL-28 melanoma cell lines (Figure 6A-C). Meanwhile, PCTAIRE1 knockdown did not induce p27 up-regulation in HaCaT keratinocytes (Figure 6D), which correlates with the lack of PCTAIRE1 dependence for growth of these non-tumorigenic cells. These results suggest that p27 accumulation induced by PCTAIRE1 depletion leads to apoptosis of melanoma cells, which is consistent with previous studies [14, 16].

### Knockdown of PCTAIRE1 suppressed tumor-forming ability *in vivo*

**Figure 7:**
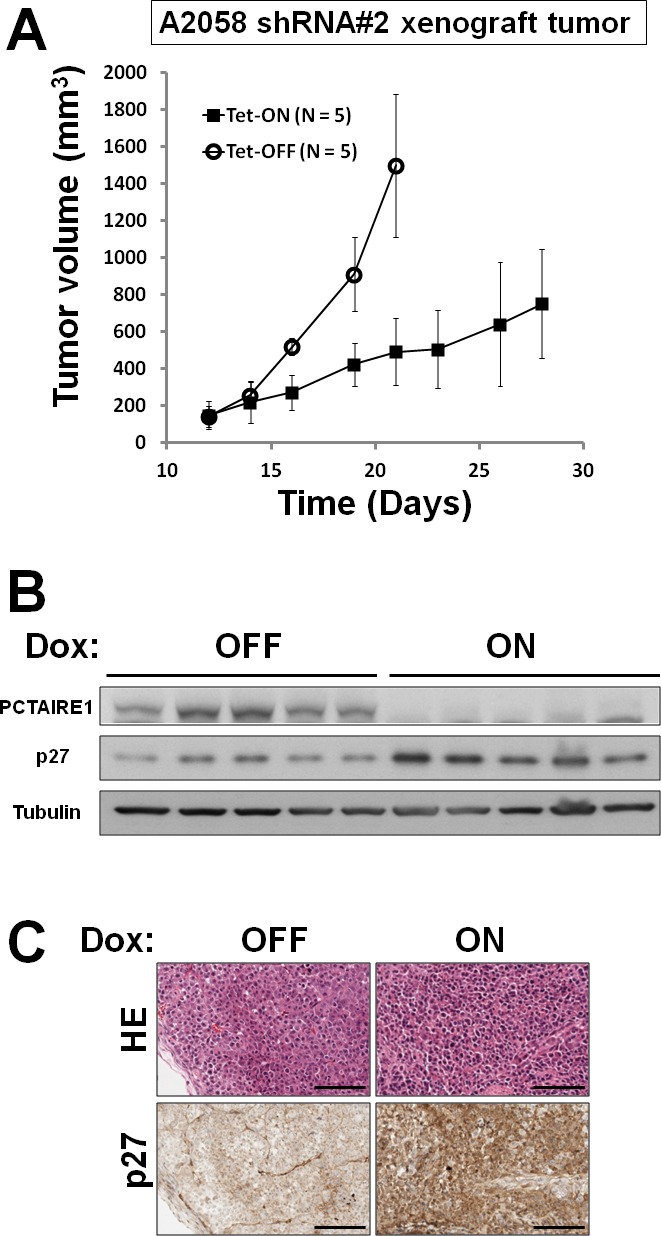
*In vivo* tumor xenograft analysis (A) A2058 cells (5.0 × 10^6^) stably containing shRNA#2 were subcutaneously injected into the flanks of *nu/nu* mice. When tumor volumes reached 150 ~ 200 mm^3^ (Day 12), the animals were provided water with (white circles) or without (black square) 2% doxycycline (“Dox”) (n = 5 mice per group). Tumor volumes (mm^3^) were measured every other day (mean ± SD; n = 5). (B) Animals were sacrificed and tumors extracted for immunoblot analyses. (C) Representative examples of hematoxylin-eosin and p27 immunostaining are provided. Scale Bar = 100 μm.

To extend these studies into an *in vivo* context, we used A2058 cells containing inducible PCTAIRE1 shRNA (#2) in a tumor xenograft model. Immunocompromised (*nu/nu*) mice were injected subcutaneously with A2058 cells, and tumors were allowed to grow for eleven days before doxycycline was added to the drinking water to induce the shRNA vector, which resulted in reduced PCTAIRE1 protein expression in tumors (Figure 7A and B). Induction of PCTAIRE1 shRNA expression remarkably suppressed tumor growth *in vivo* (Figure 7A). Meanwhile, immunoblot and immunohistochemistry analyses showed elevated levels of p27 protein in PCTAIRE1 knockdown A2058 tumor xenografts (Figure 7 B and C), consistent with our studies using cultured cells.

## DISCUSSION

**Figure 8:**
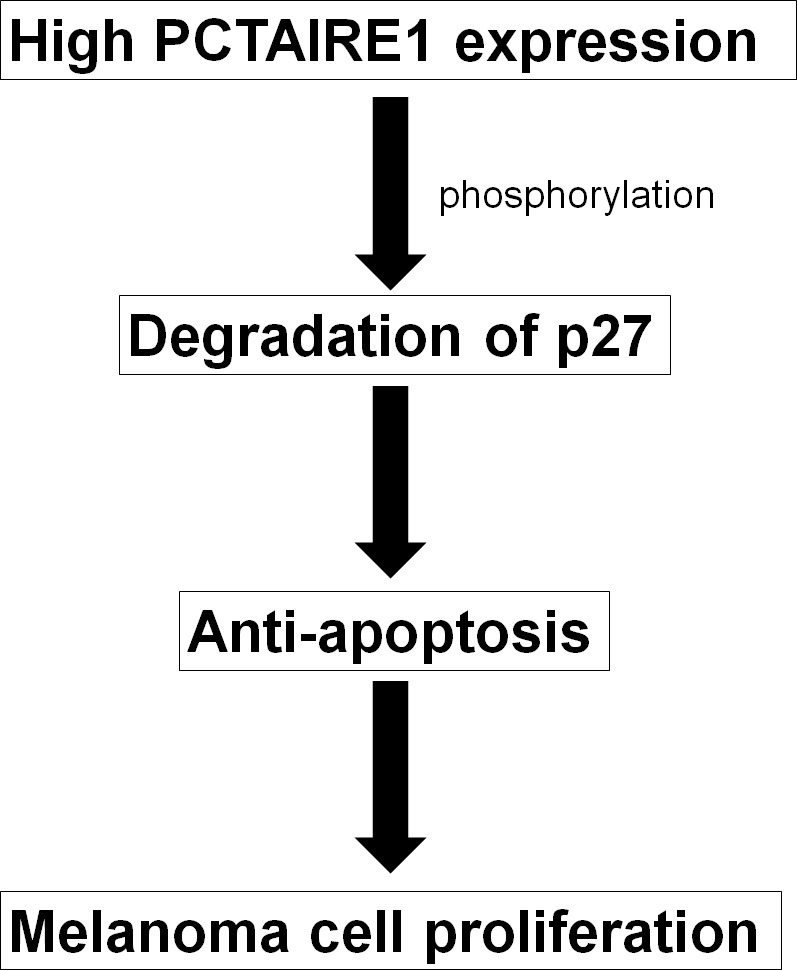
Model of PCTAIRE1 function in melanomas PCTAIRE1 promotes degradation of p27, thereby decreasing apoptosis of melanoma cells.

We report here a crucial role for the PCTAIRE family member PCTAIRE1 in melanoma cell proliferation, with PCTAIRE1 knockdown resulting in elevated p27 levels (Figure 8). p27 (also known as Kip1) is a tumor suppressor that regulates cell proliferation, motility and apoptosis [17, 18]. Consistent with its tumor suppressor role, loss of nuclear p27 is observed in metastatic melanomas and is associated with poor prognosis [19]. Decreases in p27 levels occur via increased proteosomal degradation, especially that governed by the Skp2 pathway [18, 20, 21], which is consistent with the inverse correlation of p27 and Skp2 expression levels that has been reported in human melanomas [22]. Similar to loss of p27, increased Skp2 expression levels are also correlated with poor prognosis of patients with melanoma [22]. Furthermore, Skp2 knockdown inhibits melanoma growth, suggesting that dysregulation of p27 may be a critical step in melanoma tumorigenesis [23]. In our study using melanoma cell lines, PCTAIRE1 knockdown up-regulates p27 expression, indicating that, similarly to Skp2, PCTAIRE1 plays an important role in p27 dysregulation. Further investigations including immunohistochemistry will be required to evaluate PCTAIRE1 as a novel biomarker, and whether this protein would be useful in predicting patient prognosis or treatment outcome.

Our study also shows that up-regulation of p27 led to apoptosis of melanoma cells. In our previous study, up-regulation of p27 induced by PCTAIRE1 knockdown led to late G2-M phase arrest and apoptosis in prostate, breast and cervical cancer cell lines [14]. However, melanoma cells showed no obvious cell cycle arrest following PCTAIRE1 knockdown, suggesting that the effect of PCTAIRE1 knockdown is heterogeneous among cancer cell lines. This difference is consistent with several studies on p27, in which p27 overexpression promoted G1 arrest [24], G2/M arrest or apoptosis [16] in various cancer cell lines. These studies, as well as our investigations, suggest that up-regulation of p27 alone is effective in killing cancer cells. Furthermore, p27 expression sensitizes cancer cells to other therapeutic agents [25, 26]. As such, the p27 expression level is one of the biomarkers that can be used to predict treatment outcome in cancer patients [27].

PCTAIRE1 is one of the Cdk-family kinases, which typically require cyclins for their activation. N-myristoylated cyclin Y reportedly targets PCTAIRE1 to the plasma membrane, thus stimulating its kinase activity [8]. Based on that study, we assessed the effects of siRNA-mediated cyclin Y knockdown on cell proliferation and cell death in cancer cell lines. Despite the targeting effect of cyclin Y on PCTAIRE1, cell proliferation was not suppressed by cyclin Y knockdown (data not shown), suggesting that other mechanisms that do not involve cyclin Y likely participate in PCTAIRE1 activation in cancer cells.

In summary, this work identifies a previously unrecognized role of PCTAIRE1 in regulating melanoma proliferation, and suggests that PCTAIRE1 could be an attractive target for melanoma treatments. In this context, PCTAIRE1 inhibitors could be used to restore the expression of p27, which would in turn promote the apoptosis of melanoma cells. The contribution of p27 to apoptosis is presumably only relevant to transformed cells, thus suggesting that a favorable therapeutic index may be achievable.

## METHODS

### Cell lines and cell culture

The human melanoma cell lines A2058 and SK-MEL-28, and the human immortalized keratinocyte cell line HaCaT were purchased from the American Type Culture Collection or cell line service. Cell lines were cultured in RPMI or DMEM supplemented with 10% FBS.

### Reagents and antibodies

Pre-designed small interfering RNA (siRNA) directed against human PCTAIRE1 (siRNA#1:1472, siRNA#2:1656) and negative scramble control (#1, #2), were purchased from Life Technologies. Antibodies against PCTAIRE1 (rabbit: HPA001366, Sigma), p27 (mouse G173-524: BD, or rabbit C-19: Santa Cruz), alpha-tubulin (T5168, Sigma) and horseradish-peroxidase (HRP)-conjugated secondary antibodies (GE Health Care) were purchased from the indicated sources.

### Gene expression

Individual cancer data sets were downloaded from Oncomine (https://www.oncomine.org) as previously described [14].

### RNA interference

For transient knockdown, cells were transfected with siRNA duplexes by a reverse transfection method using Lipofectoamine RNAiMAX according to the manufacturer's instructions (Life Technologies).

### Tet-inducible short hairpin RNA constructs, lentivirus and infection

PCTAIRE1 shRNA#1 (GCTCTCATCACTCCTTCACTT), PCTAIRE1 shRNA#2 (GACCTACATTAAGCTGGACAA) and scramble-control (CAACAAGATGAAGAGCACCAA) were cloned into the inducible pLKO-Tet-On puromycin vector as previously described [28]. Lentiviral supernatants were generated according to an established protocol [28]. Cells were selected with 2 μg/ml puromycin (MP Biomedicals) and expanded. Induction of shRNA was achieved by the addition of 100 ng/ml doxycycline (Sigma) to the medium.

### Cell viability assays using ATP measurement

Cell Titer Glo (Promega) was used for cell viability estimation. Cells were plated at a density of 5,000~10,000 cells per well in 100 μL and cultured for 48 or 72 hours with or without treatments. Plates were then removed from the incubator and allowed to equilibrate to room temperature for about 10 minutes. Cell Titer Glo solution was added at 100 μl per well and the plates were kept in the dark for 15 minutes before reading the luminescence with a luminometer (Luminoskan Ascent; Thermo Scientific Corporation) and a 1-sec integration time per sample.

### Cell cycle analysis by FACS

Cells were first fixed with cold 70% ethanol and then treated with propidium iodide (20 μg/ml) and ribonuclease A (10 μg/ml) prior to cell cycle analysis using a FACS Canto apparatus (Becton Dickinson). A total of 30,000 events were analyzed.

### Analysis of apoptosis

Cells were processed using an annexin V-PI apoptosis assay kit (FITC-annexin V/ propidium iodide staining) according to the manufacturer's protocol (Life Technologies). A total of 20,000 events were analyzed by flow cytometry.

### Extraction of total RNA and quantitative RT-PCR analysis

Total RNA was isolated from cultured cells using the RNeasy Plus Mini kit (Qiagen). We reverse-transcribed RNA using Superscript III according to the manufacturer's instructions (Life Technologies) and complementary DNA samples were analyzed by the SYBR green system (Promega). The sequences for the primers are as follows:

Human PCTAIRE1

Forward: 5′- GCAGTGACCCTGGAGAGG -3′

Reverse: 5′- TCAAGTCCTCGTGCACAATC -3′

Human GAPDH

Forward: 5′- GAAGGTGAAGGTCGGAGTC -3′

Reverse: 5′- ATGGGATTTCCATTGATGAC -3′

All experiments were performed in duplicate and normalized with respect to GAPDH levels.

### SDS-PAGE and immunoblotting

Cells were washed twice with PBS and harvested with radioimmunoprecipitation assay (RIPA) buffer composed of 20 mM Tris-HCl, pH 7.5, 150 mM NaCl, 0.1mM EDTA, 1% Nonidet P-40, 0.1% SDS, 5 mM NaF and an EDTA-free cOmplete protease cocktail tablet (Roche). Cells were left on ice for 20 min and centrifuged at 14,000 × *g* for 10 minutes. The Bio-Rad protein assay kit (Bio-Rad) was used to determine protein concentrations. Proteins were separated on SDS-PAGE 4-15% gradient gels (Life Technologies) and transferred onto nitrocellulose membranes (Bio-Rad). Membranes were blocked for 1 hour in Tris-buffered saline (TBS) with 0.05% Tween-20 and 5% non-fat dry milk, and then incubated overnight at 4 °C with primary antibodies diluted in blocking buffer. Membranes were rinsed three times in TBS with 0.05% Tween-20 and incubated with secondary HRP-conjugated antibodies for 1 hour at room temperature. An enhanced chemiluminescence (ECL) method (GE Health Care) was used for detection.

### Cell growth assay

To measure cell growth rates, 1.0 × 10^5^ cells with Tet-inducible shRNA targeting PCTAIRE1 were plated onto 60 mm diameter plates. One day later, the culture media was changed to that with (ON) or without (OFF) doxycycline (100 ng/ml). The numbers of cells were counted 1, 3, 5, and 7 days after seeding using the Countess automated cell counter (Life Technologies). In siRNA experiments, 2.0 × 10^5^ cells were reverse-transfected with siRNAs targeting PCTAIRE1 or scramble control. After 3 days, the number of cells was counted.

### Clonogenic assay

Cells with Tet-inducible shRNA targeting PCTAIRE1 were seeded at 300~1,000 cells per well in 6 well (35 mm) dishes. Cells were cultured with (ON) or without (OFF) doxycycline for 10~14 days before fixing and staining. After fixation by methanol, cells were washed with PBS and incubated with 0.5% crystal violet dye in 25% methanol for 15 minutes. A colony was defined as consisting of at least 50 cells.

### Tumor xenograft experiments

All animal experiments were approved by the IACUC of the Sanford-Burnham Medical Research Institute. A2058 cells (5.0 × 10^6^) resuspended in 200 μl PBS were injected subcutaneously into the flanks of *nu/nu* mice. Tumor volumes were calculated using the following formula: (long axis × short axis^2^)/2.

### Immunohistochemistry

Dewaxed tissue sections (4.0-5.0 μm) were immunostained as reported previously [29] using a mouse monoclonal antibody to p27. Application of the primary antibody was followed by incubation with goat anti-mouse polymer-based EnVision-HRP-enzyme conjugate (DakoCytomation). DAB chromogen (DakoCytomation) was applied to yield a brown color.

### Statistical Analysis

Means and standard deviation (SD) were calculated statistically from three determinations. The data are expressed as mean ± SD. The statistical significance of differences between various samples was determined by Student's t-test. p < 0.05 was considered significant.

### Disclosure

John C. Reed is an employee of the Roche Group, AG. The other authors have no financial conflict of interest.
